# Incidence and prognostic factors in severe drug-induced interstitial lung disease caused by antineoplastic drug therapy in the real world

**DOI:** 10.1007/s00432-022-03932-3

**Published:** 2022-02-07

**Authors:** Sawako Kaku, Hidehito Horinouchi, Hirokazu Watanabe, Kan Yonemori, Takuji Okusaka, Narikazu Boku, Naoya Yamazaki, Akira Kawai, Yuichiro Ohe, Masahiko Kusumoto

**Affiliations:** 1 Department of Diagnostic Radiology, National Cancer Center Hospital, 5-1-1 Tsukiji, Chuo-ku, Tokyo, 104-0045 Japan; 2 Course of Advanced Clinical Research of Cancer, Juntendo University Graduate School of Medicine, Tokyo, Japan; 3 Department of Thoracic Oncology, National Cancer Center Hospital, 5-1-1 Tsukiji, Chuo-ku, Tokyo, 104-0045 Japan; 4 Department of Medical Oncology, National Cancer Center Hospital, 5-1-1 Tsukiji, Chuo-ku, Tokyo, 104-0045 Japan; 5 Department of Hepatobiliary and Pancreatic Oncology, National Cancer Center Hospital, 5-1-1 Tsukiji, Chuo-ku, Tokyo, 104-0045 Japan; 6 Department of Gastrointestinal Medical Oncology, National Cancer Center Hospital, 5-1-1 Tsukiji, Chuo-ku, Tokyo, 104-0045 Japan; 7 Department of Dermatologic Oncology, National Cancer Center Hospital, 5-1-1 Tsukiji, Chuo-ku, Tokyo, 104-0045 Japan; 8 Department of Musculoskeletal Oncology, National Cancer Center Hospital, 5-1-1 Tsukiji, Chuo-ku, Tokyo, 104-0045 Japan

**Keywords:** Drug-induced interstitial lung disease, Cancer, Thoracic cancer, Lung cancer, Malignant melanoma, Antineoplastic drug therapy, Chemotherapy, Molecular targeted drug therapy, Immune-checkpoint inhibitor, Diffuse alveolar damage

## Abstract

**Purpose:**

Investigate the frequency and prognostic factors of severe drug-induced interstitial lung disease (DILD) caused by antineoplastic drugs regardless of cancer types or type of drugs.

**Methods:**

From 2014 to 2018, we reviewed patients with a history of antineoplastic agents administration in the real-world database of our hospital's electronic medical record and extracted patients who experienced "severe" DILD, requiring hospitalization with treatment or developed during hospitalization and required treatment. We collected patients' backgrounds, clinical and radiological features, laboratory data, treatment, and survival outcomes.

**Results:**

19,132 cancer patients received antineoplastic drug therapy during the study period, and 120 (0.62%) experienced severe DILD. The incidence of severe DILD in patients with thoracic cancer was highest among the patients included in this analysis (2.52% vs. 0.34% other cancers). Diffuse alveolar damage (DAD) pattern on CT was associated with higher mortality in patients with severe DILD compared with non-DAD pattern (hazard ratio [HR], 11.24; 95% CI, 4.82–26.2). Multivariate analysis revealed that the DAD pattern at diagnosis as severe DILD (HR, 3.59; 95% CI, 1.17–11.03), concurrent/previous interstitial lung disease (HR, 3.20; 95% CI, 1.27–8.10), and ECOG performance status of 2–4 (HR, 3.81; 95% CI, 1.10–13.17) were independent risk factors for mortality in patients with severe DILD.

**Conclusions:**

The frequency of severe DILD was highest in patients with thoracic cancer. The DAD pattern was associated with a poor outcome. From the perspective of DILD, special attention should be paid when administering antineoplastic agents to patients with thoracic cancer.

## Introduction

Drug-induced interstitial lung disease (DILD), also known as drug-induced pneumonitis or drug-induced pulmonary toxicity, is a significant treatment-related complication in cancer treatment (Sakurada et al. 2015; Leger et al. 2017). DILD is known to have substantial adverse clinical consequences in severe cases, including treatment-related death (Ando et al. 2006). A population-based study reported an incidence of respiratory failure attributable to drug-induced interstitial lung disease of 6.6 per 100,000 patient-years, with more than half of the cases associated with chemotherapeutic agents (Dhokarh et al. 2012).

Many clinical trials and postmarketing surveillance referred to DILD as a potential adverse event or side effect and have examined both pathogenic and prognostic factors (Gemma et al. 2020; Osawa et al. 2015; Abdel-Rahman and Elhalawani 2015). Additionally, the use of drugs with new action mechanisms, such as molecular targeting agents and immune checkpoint inhibitors (ICIs), has increased the frequency of DILD (Johkoh et al. 2021). Among cancer-specific reports, chemotherapy-related pneumonitis reportedly occurs in approximately 30% of lung cancer patients and is believed to be the most common cause of treatment-related death (Minami-Shimmyo et al. 2012).

However, no real-world studies have investigated cross-sectional incidences and prognostic factors in drug-induced interstitial lung disease regardless of cancer or drug. Additionally, the number of severe drug-induced interstitial lung disease cases is small, making it challenging to collect data. Thus, we investigated the incidence, mortality, and prognostic factors of severe DILD in cancer treatment, regardless of drug class or tumor type.

## Materials and methods

### Patient selection and data collection

We reviewed the medical records of patients who received antineoplastic drug therapies and extracted patients experiencing severe DILD retrospectively. To identify the incidence of severe DILD, we first collected data from all the patients who received antineoplastic drug therapy at our hospital during the study period. We extracted 19,132 patients with a history of antineoplastic drug administration in the real-world database of our hospital's electronic medical record. These data included patient age, sex, type of cancer, and the antineoplastic drugs administered. We then reviewed patients whose CT scan reports contained drug-induced lung injury, interstitial pneumonia, or similar terms during the study period. Next, we identified patients with newly appearing interstitial findings in the lungs, including drug-induced interstitial pneumonitis. Finally, we selected patients with "severe" drug-induced lung injury according to selection/exclusion criteria. The selection criteria for "severe" DILD were as follows: (1) patients receiving antineoplastic drugs, (2) patients repeating regular thoracic CT scans, (3) patients developing drug-induced interstitial lung disease because of antineoplastic drugs required hospitalization, and (4) patients developing drug-induced interstitial lung disease because of antineoplastic drugs during hospitalization.

The exclusion criteria for severe DILD were as follows: (1) patients with DILD caused by investigational drugs, (2) patients with DILD developed by treatments other than antineoplastic drugs, (3) patients who developed DILD because of antineoplastic drugs and hospitalized for observation, and (4) patients who developed DILD because of antineoplastic drugs during hospitalization without additional treatment. In evaluating DILD imaging patterns, two board-certified radiologists (M.K. and H.W.) independently reviewed chest computed tomography (CT) images obtained at the time of severe DILD and classified according to CT image pattern definitions^5.^ The imaging patterns of ILD are as follows: (1) diffuse alveolar damage (DAD) pattern; (2) hypersensitivity pneumonia (HP) pattern; (3) organizing pneumonia (OP) pattern; and (4) others. In addition, we collected the data of patients' backgrounds, types of antineoplastic drugs administered, and other clinical information, including outcomes.

### Statistical analysis

Survival time was defined as the period from the onset of severe DILD until death or censoring. Cancer-specific death was defined as death after recovery from severe DILD. As for the overall survival analysis, we used the Kaplan–Meyer estimate and the Cox proportional hazard model. We considered cancer-related death as a competing risk for DILD-related death in this analysis. The cumulative incidence of DILD-related deaths was calculated using a cumulative incidence function, taking into account competing risks. The Fine-Gray hazard model was used to identify prognostic factors of severe DILD to adjust for the competing risk (Fine and Gray 1999). The factors used in the univariate and multivariate analyses were as follows: the type of cancer, history of thoracic surgery, smoking history, history of irradiation to the chest, concurrent/previous interstitial lung disease including drug-induced interstitial lung disease, ECOG performance status (PS) on admission, oxygen requirement on admission, the existence of emphysema, patterns of interstitial changes in the lungs (DAD pattern, OP pattern, HP pattern, and others), and use of steroid pulse therapy. Statistical significance was set at *P* < 0.05. All statistical analyses were performed using EZR version 1.53 (Saitama Medical Center, Jichi Medical University, Saitama, Japan) and a graphical user interface (R Foundation for Statistical Computing, Vienna, Austria).

## Results

### The incidence and patient characteristics of severe DILD

**Fig. 1:**
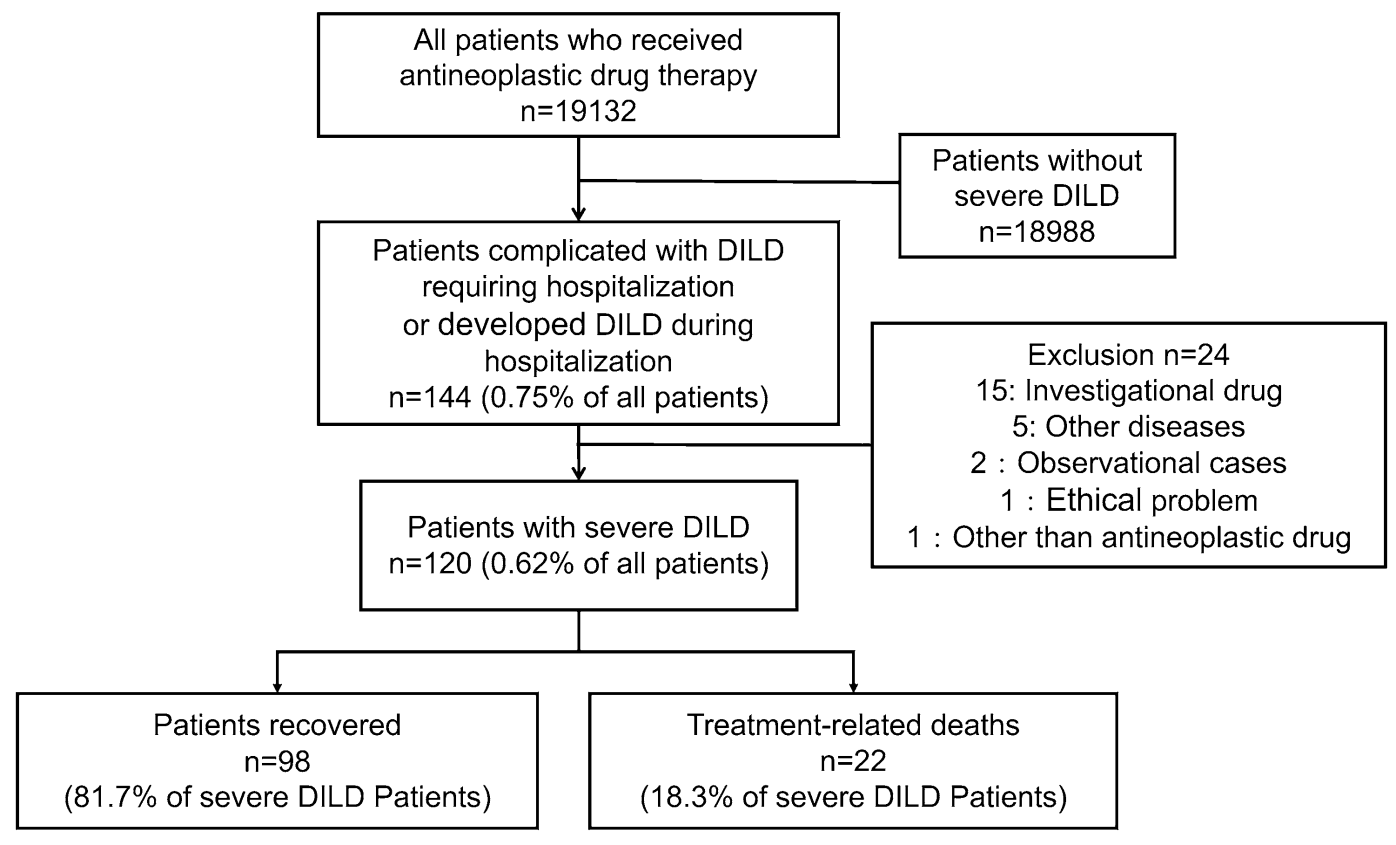
Patient flow diagram

From 2014 to 2018, a total of 19,132 patients were treated with antineoplastic drugs at our institution; of these patients, 120 (0.62%) developed severe DILD (Fig. 1).

**Fig. 2:**
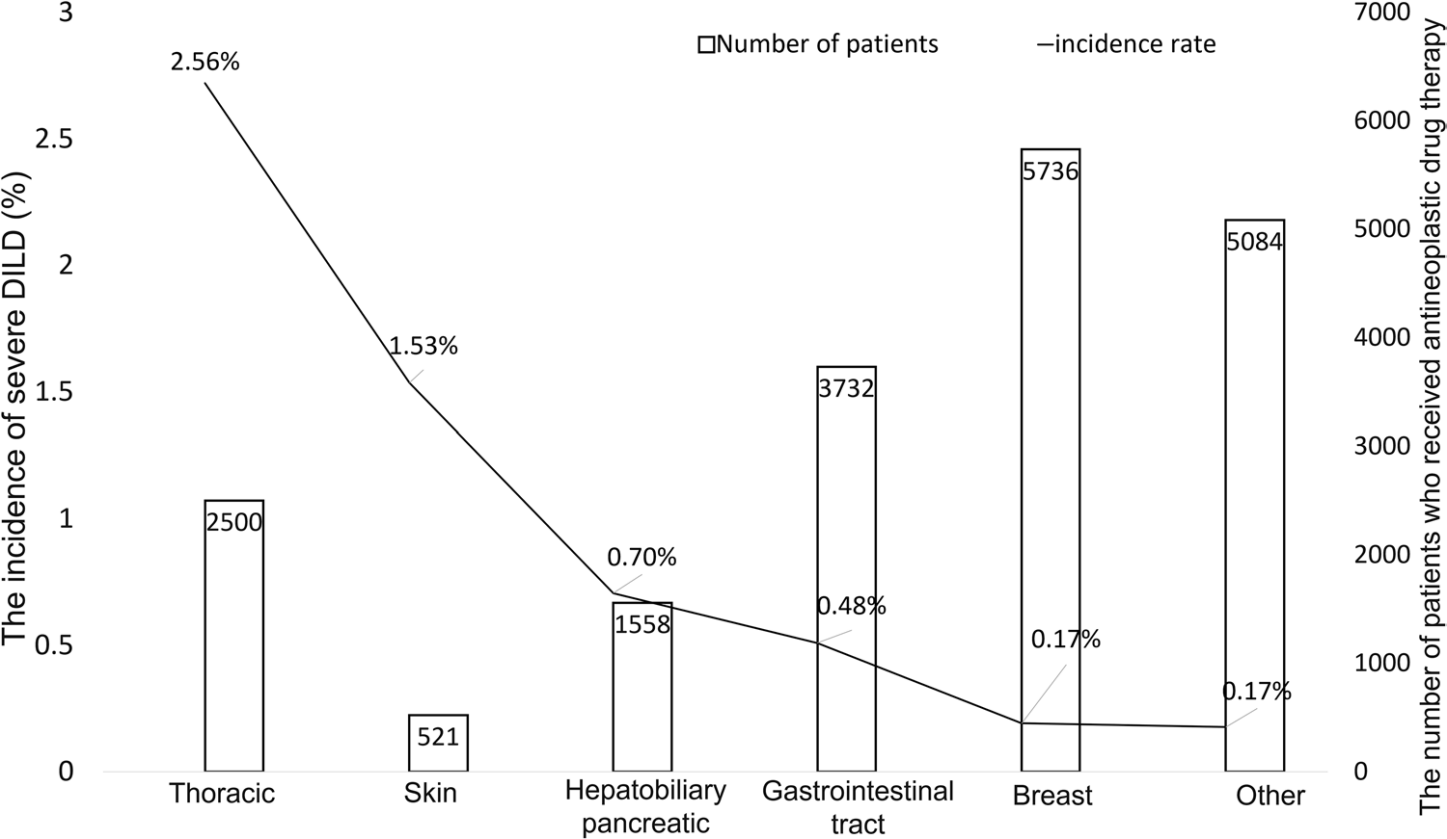
The incidence rate of severe DILD and the number of patients who received antineoplastic drug therapy

The incidence of severe DILD among all patients who received antineoplastic drugs was highest for patients with thoracic cancer (2.52%), taking the frequency of each type of cancer into consideration. Skin malignancy (1.53%) and hepatobiliary cancer (0.70%) had the second and third highest incidences of severe DILD following thoracic cancer (Fig. 2). The characteristics of the patients with severe DILD are shown in Table 1. The median age was 66 years (range: 25–86 years), and the proportion of males was higher than that of females (70.0% vs. 30.0%). Thoracic cancer accounted for more than half of the primary organs (53.3%). The following most common sites of primary cancer were hepatobiliary and pancreatic cancer (9.2%), breast cancer and upper gastrointestinal cancer (8.3%), lower gastrointestinal cancer, and melanoma (6.7%). The majority of the patients' PS scores were 1 (55.1%) or 2 (22.9%). Among patients who developed severe DILD, 15 (12.5%) had concurrent/previous interstitial lung disease, 62 (53.3%) had a smoking history, and 18 (15.0%) had a history of thoracic radiotherapy. In addition, 91 (75.8%) required oxygen supplements, and 60 (50.0%) were treated by steroid pulse therapy. The data cut-off was March 31, 2019. The median follow-up time from the onset of severe DILD was 134.5 days (range: 1–1229 days).

**Table 1:** Patient characteristics

Patient backgrounds	*N* = 120	
Age mean (range)	66	(25–86)
Sex	*N*	%
Male	84	70.00
Female	36	30.00
Etiology of cancer		
Lung	64	53.30
Biliary tract and pancreas	11	9.20
Breast	10	8.30
Upper GI^a^	10	8.30
Lower GI	8	6.70
Melanoma	8	6.70
Other	9	7.50
Performance status		
0	6	5.10
1	65	55.10
2	27	22.90
3	14	11.90
4	6	5.00
Unknown	2	1.60
Number of regimen (mean, range)	3.0 [1.00- 9.00]	
Concurrent/previous ILD^b^	15	12.50
Smoking history	62	53.30
Operation history	24	20.00
Radiation history	18	15.00
Oxygen supplementation	91	75.83
Steroid pulse therapy	60	50.00

^a^*GI* gastrointestinal

^b^*ILD* interstitial lung disease

### The outcome of severe DILD caused by antineoplastic drugs

The mortality rate in all 126 patients who developed severe DILD was 18.3% (*n* = 23) (Table 2). 98 patients recovered, and over half of them (99 patients: 74.2%) required the continued use of corticosteroids to maintain the resolution of DILD. Concerning the type of cancer treatment, the highest mortality rate was observed among cases of severe DILD caused by conventional cytotoxic agents. In detail, the proportions of fatal outcomes were as follows: cytotoxic agent, 21.1% (16/76); molecularly targeted agent, 9.5% (2/21); and ICIs, 17.4% (4/23). Patients receiving ICIs were the least likely to achieve steroid withdrawal after the onset of severe DILD (13.0%). All the severe DILD-related deaths arising from the use of molecularly targeted agents occurred in patients receiving epidermal growth factor receptor (EGFR) tyrosine kinase inhibitors (TKIs). Among the severe DILD patients receiving PD-1 inhibitors, 3 out of 23 patients (13.0%) died. Of these 3 deaths, 1 patient had received combination therapy with a PD-1 inhibitor and CTLA-4 inhibitor. In contrast, among the 65 patients who received PD-L1 inhibitors and developed severe DILD, only one patient (1.54%) died.

**Table2:** Incidence and outcome of severe DILD caused by antineoplastic drugs

	Number of patients	Number of drugs	Severe DILD with hospitalization		Imaging pattern of DILD		Successful steroid withdrawal		Number of treatment-related death in severe DILD	
			*N*	%			*N*	%	*N*	%
Total	19,132	56,651	120	0.62			31	25.83	22	18.33
Cytotoxic agents	17,251	51,083	76	0.44	HP	34	23	30.26	16	21.05
					DAD	13				
					OP	12				
					other	17				
Molecular targeted agents	3989	5385	21	0.52	HP	5	6	28.57	2	9.52
					DAD	1				
					OP	12				
					Other	2				
					no CT	1				
Immune-checkpoint inhibitors	975	1165	23	2.35	HP	8	3	13.04	4	17.39
					DAD	1				
					OP	11				
					Other	2				
					no CT	1				

### CT pattern of severe DILD caused by antineoplastic drugs

The most common CT pattern in patients with severe DILD was the HP pattern (39.2%). The OP pattern (29.1%) and the DAD pattern (12.5%) were the second and third most common. The mortality rate of patients with severe DILD was highest for those with the DAD pattern (53.3%), followed by those with an OP pattern (11.4%) and those with an HP pattern (6.4%). The proportion of patients who could discontinue corticosteroids after severe DILD was inversely associated with the mortality rate (Table 3). The HP pattern was commonly observed in patients who had received gemcitabine, docetaxel, nivolumab, and irinotecan. The severe DILD patients with an HP pattern had the highest steroid withdrawal rate (31.9%). The OP pattern of severe DILD was frequent among patients treated by pembrolizumab and nivolumab. Paclitaxel was the most common drug suspected of causing the DAD pattern in patients with severe DILD, and none of these patients were able to withdraw from steroid treatment.

**Table 3:** CT pattern of severe DILD caused by antineoplastic drugs

Imaging pattern of DILD	*N*	%	Suspected drug	*N*	Number of treatment-related deaths and mortality (%) in patients with severe DILD		Successful steroid withdrawal (%)
HP	47	39.17			3	(6.38)	15 (31.91)
			Gemcitabine	8	1	(11.11)	
			Paclitaxel	7	0	(0.00)	
			Nivolumab	6	0	(0.00)	
			Irinotecan	4	0	(0.00)	
			Docetaxel	3	0	(0.00)	
			Osimertinib	3	0	(0.00)	
			Pembrolizumab	3	0	(0.00)	
			Other	13	2	(15.38)	
OP	35	29.17			4	(11.43)	9 (25.71)
			Pembrolizumab	7	1	(14.29)	
			Nivolumab	4	2	(50.00)	
			Amrubicin	3	0	(0.00)	
			Afatinib	3	0	(0.00)	
			Other	18	1	(5.56)	
DAD	15	12.50			8	(53.33)	0(0.00)
			Paclitaxel	4	2	(50.00)	
			Pemetrexed	2	1	(50.00)	
			Other	9	5	(55.56)	
Other	21	17.50		21	6	(28.57)	7 (33.33)
No CT	2	1.67		2	1	(50.00)	0(0.00)

### Cumulative incidences and mortality of DILD

**Fig. 3:**
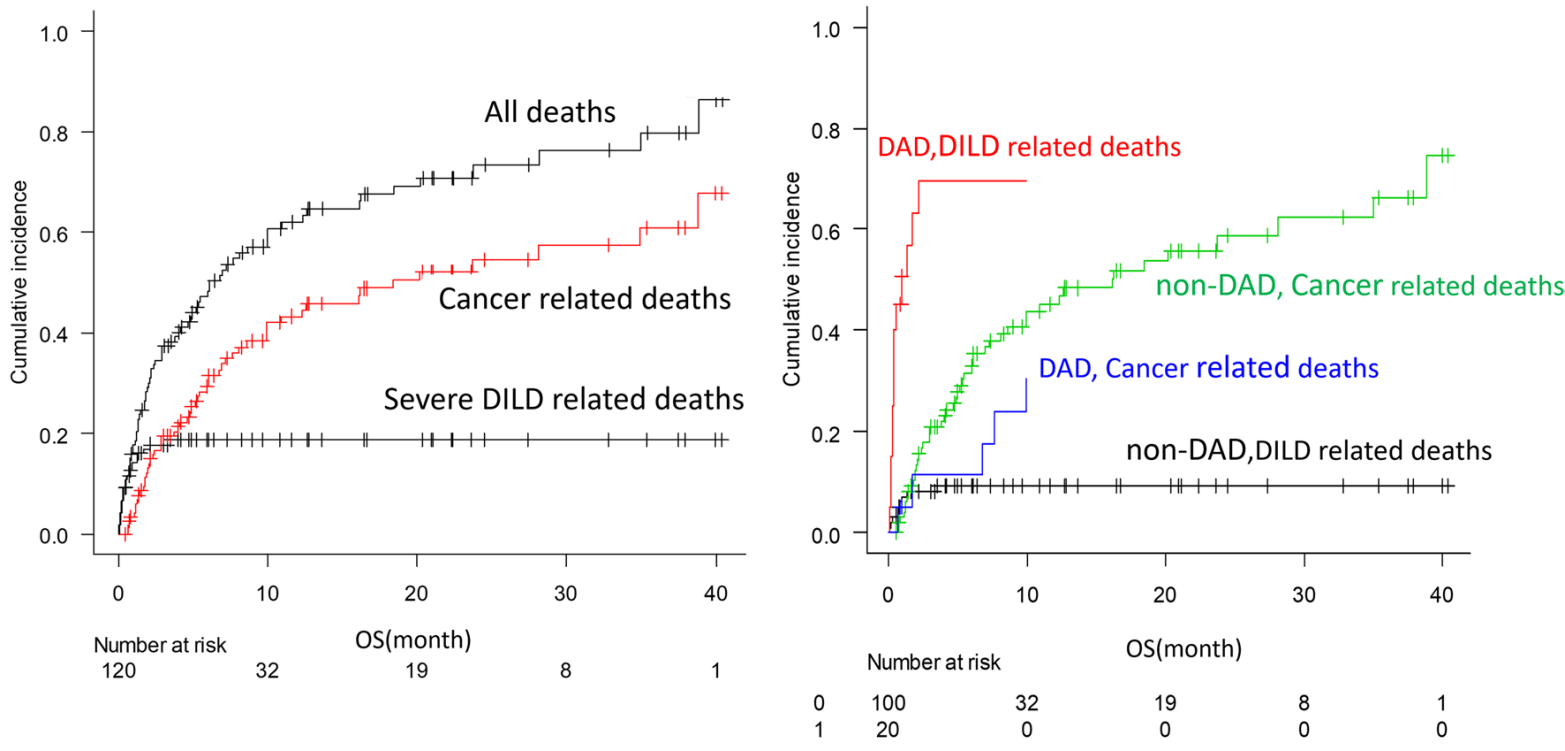
**a** Cumulative incidence of severe DILD-related death, cancer death, and all death. **b** Cumulative incidence of DILD and cancer deaths classified by presence or absence of DAD pattern

In Fig. 3a, the cumulative incidence of severe DILD-related deaths, taking cancer-related deaths into account as a competing risk, was shown. The mortality rate was higher in the group with the DAD pattern at the onset of severe DILD (DAD vs. non-DAD: 53.3% vs. 13.3%). The DAD pattern was associated with increased mortality in patients with severe DILD (hazard ratio [HR], 11.24; 95% CI, 4.82–26.2). The median survival time in patients with severe DILD with the DAD pattern was 10 days (range: 2–65 days) (Fig. 3b).

### Prognostic factors of severe DILD

In univariate analyses, male sex, a history of thoracic surgery, a concurrent/previous interstitial lung disease, a history of smoking, ECOG PS of 2 or more, and the DAD pattern were associated with a higher risk of severe DILD-related death. Multivariate analysis showed that an ECOG PS of 2 or more (HR, 3.81; 95% CI, 1.10–13.17), the DAD pattern (HR, 3.59; 95% CI, 1.17–11.03), and concurrent/previous interstitial lung disease (HR, 3.20; 95% CI, 1.27–8.10) were independent prognostic factors (Table 4).

**Table 4:** Univariate and multivariate analyses of prognostic factors of severe DILD

Factors	Univariate analysis			Multivariate analysis		
	Hazard ratio	95% CI	*P* value	Hazard ratio	95% CI	*P* value
Sex						
Male	1	0.01–0.73	0.02	1	0.05 –3.6	0.42
Female	0.09	0.44				
History of lung surgery						
Absence	1	1.05–5.66	0.04	1	0.59–5.21	0.31
Presence	2.44	1.76				
History of interstitial lung disease						
Absence	1	2.24–12.67	< 0.01	1	1.27–8.10	0.01
Presence	5.33	3.2				
Smoking history						
Absence	1	1.18–22.63	0.03	1	0.23–6.88	0.78
Presence	5.18	1.26				
Performance status						
0–1	1	2.32–16.46	< 0.01	1	1.10–13.17	0.03
≥ 2	6.18	3.81				
DAD pattern on CT						
Absence	1	4.82–26.20	< 0.01	1	1.17–11.03	0.02
Presence	11.24	3.59				

## Discussion

To our knowledge, this is the first report to reveal the incidence and prognostic factor of severe DILD among the most significant number of patients who received antineoplastic drug therapy irrespective of cancer types or drug types. We examined 19,132 consecutive patients who received antineoplastic drug therapy and underwent regular thoracic CT examinations. In summary, thoracic cancer had the highest number (*n* = 64) and incidence rate (2.56%) of severe DILD. The incidence and mortality of severe DILD varied among the types of antineoplastic drugs. In addition, the DAD pattern, concurrent/previous interstitial lung disease, and an ECOG PS of 2 or more were identified as independent prognostic factors of severe DILD. These findings help to predict treatment-related deaths caused by DILD regardless of the type of malignant neoplasm or administered antineoplastic drug therapy.

Our study highlighted that the incidence of severe DILD was highest among patients with thoracic cancer. The incidence of severe DILD in patients with thoracic cancer was more elevated than patients with other cancers (2.5% vs. 0.3%). In the previous reports, the incidence of DILD during cancer drug therapy in non-small cell lung cancer was reported to be 5% (Fujimoto et al. 2016). The incidence of drug-induced interstitial lung disease in thoracic cancer patients in our study was similar. This tendency was consistent throughout the types of antineoplastic drugs administered, such as cytotoxic agents, molecular target agents, and ICIs. Regarding the incidence and severity of DILD in cross-sectional analyses covering multiple types of cancers or treatments, a large retrospective cohort study examining gemcitabine-related DILD reported that patients with lung cancer were more likely to develop DILD than patients with other cancers (Hamada et al. 2016). Among patients receiving combination therapy consisting of docetaxel and gemcitabine, lung cancer patients also had a higher risk of developing severe DILD than breast cancer patients (Binder et al. 2011).

Additionally, severe DILD in patients with cancers other than thoracic cancer and skin malignancy were almost limited to patients treated with cytotoxic agents. Patients with NSCLC had a higher incidence of grade 3 or higher pneumonia than patients with melanoma (1.8% vs. 0.2%; *P* < 0.001) (Nishino et al. 2016), while skin malignancies had the second-highest incidence of severe DILD, and all the patients with melanoma who developed DILD had been treated with ICI. In a meta-analysis of drug-induced interstitial lung disease with PD-1 inhibitors, the incidence of grade 3 pneumonia in patients treated with PD-1 inhibitors was 0.2%. Patients with thoracic cancer are more susceptible to severe drug-induced interstitial lung disease because of the tumor burden and the presence of background lung injuries, such as interstitial lung abnormalities or emphysema (Bouros et al. 2002; Toh et al. 2004). Our results demonstrate the different backgrounds and susceptibilities of patients who develop severe DILD among patients with various tumors and treated with various antineoplastic drugs.

Concurrent/previous interstitial lung disease (ILD) and a PS of 2–4 were identified as predictors of a poor prognosis after the onset of severe DILD. Although these factors have been previously detected as risk factors for the development of DILD (Minami-Shimmyo et al. 2012; Hamada et al. 2016; Tirumani et al. 2015; Nakagawa et al. 2012), a few studies have examined them as prognostic factors of DILD. Pre-existing ILD and a decreased PS have been identified as prognostic factors in postmarketing surveys of erlotinib and a meta-analysis of 24 phase III clinical trials of EGFR-TKIs (Gemma et al. 2014; Qi et al. 2015). Our present study showed that pre-existing ILD and a decreased PS could be considered prognostic factors of severe DILD even when all antineoplastic drugs were comprehensively considered. We confirmed that these risk factors for developing DILD affect prognosis, since our study focused on severe cases, and our findings underscore the importance of these factors. Given the potentially fatal outcome of DILD, patients receiving any antineoplastic drugs who have these predictors of a poor prognosis should be managed carefully throughout their treatments.

Among the HRCT imaging findings, the DAD pattern was an independent prognostic factor in severe DILD patients. The mortality rate was significantly higher in the group with the DAD pattern (DAD, 53.3% vs. non-DAD, 13.3%). Among the previous reports, only one observational study examining nivolumab-induced DILD reported the DAD pattern as a statistically significant predictor of a poor prognosis (Saito et al. 2020). The high mortality rate in patients with the DAD pattern agrees with the findings of previous reports on DILD (40–83.3%) (Gemma et al. 2014, 2019, 2020; Shi et al. 2014; Hotta et al. 2010; Tomii et al. 2017). A propensity of certain drugs to cause DAD was not identified in our study. Nevertheless, this study reviewed and analyzed a sufficient number of severe DILD cases. The results emphasize that patients with the DAD pattern have a high mortality rate and a poor prognosis regardless of the type of cancer or antineoplastic drug treatment.

The present study had some limitations. First, admission criteria vary among physicians, and there is a possibility that the severity of DILD may not be consistent. CTCAE ver. 5. defines Grade 3 pneumonia as "Severe symptoms; limiting self-care ADL; oxygen indicated." More than 70% of patients in our patient population required oxygen demand, and all patients were receiving steroids. Additionally, since we excluded observational cases, our definition of "severe" DILD is somewhat similar to CTCAE grade 3 pneumonitis. Second, the study was performed retrospectively at a single institution. However, severe DILD is not frequently encountered in clinical practice, and the progress of multiple cases can be challenging to follow in detail. Nevertheless, our institution has a large caseload of patients requiring cancer medications. We reviewed the drug histories of all the patients who received antineoplastic drugs, and board-certified diagnostic radiologists reviewed the CT images of patients who developed severe DILD. Thus, the consecutiveness of cases is authentic, to some extent. Finally, we could not obtain pathological diagnoses in most of the cases. A previous report has described a concordance between HRCT interpretation and tissue diagnosis of about 50% in patients with DILD (Cleverley et al. 2002). This result supports the use of HRCT as a non-invasive test to predict prognosis, to some extent, when a bronchoscopic alveolar lavage or biopsy cannot be performed.

In conclusion, across all cancer types and all antineoplastic agents, the frequency of DILD was highest among patients with thoracic cancer. In addition, the DAD pattern, concurrent/previous ILD, and a PS of 2–4 were associated with a poor outcome. Therefore, from the perspective of DILD, a special attention should be paid to the use of antineoplastic agents in patients with thoracic cancer and other patients with the factors mentioned above.

## Data Availability

The datasets generated during the current study are not publicly available due to ethical restrictions, but are available from the corresponding author on reasonable request.
